# Real-world evaluation of an OCT-based AI decision-support system for neovascular AMD activity triage in teleophthalmology

**DOI:** 10.3389/fopht.2026.1870572

**Published:** 2026-07-01

**Authors:** Kai Rothaus, Michael Grün, Henrik Faatz, Martin Ziegler, Georg Spital, Albrecht Lommatzsch, Matthias Gutfleisch, Britta Heimes-Bussmann, Clemens Lange

**Affiliations:** 1 Department of Ophthalmology, St. Franziskus Hospital, Münster, Germany; 2 Eye Centre, Freiburg Medical Centre, University of Freiburg, Freiburg, Germany

**Keywords:** age-related macular degeneration, artificial intelligence, deep learning, deepeye^®^, diagnostic accuracy, OCT, telemedicine

## Abstract

**Purpose:**

To evaluate real-world agreement between a CE-marked OCT-based AI decision-support system and routine retreatment decisions for neovascular AMD within a teleophthalmology workflow.

**Methods:**

Retrospective clinical study including 429 OCT examinations from 247 patients (306 treated eyes) with neovascular AMD. Retinal specialists made routine retreatment decisions (“inject” vs “watch-and-wait”) using full clinical context. Independently, the AI system (deepeye^®^ TPS, version 1.2) analyzed the current OCT volume only (no prior OCT, visual acuity, treatment interval, or clinical notes) and generated a Disease Activity Score (DAS; 0–100) used to derive an “inject” vs “watch-and-wait” recommendation. Discrepant cases were re-evaluated by senior graders to establish a double-senior-graded (DSG) reference standard. Implementation analyses assessed a deferral (“safety zone”) strategy. Main outcome measures included agreement/accuracy, sensitivity, and specificity versus real-world decisions and the double-senior-graded (DSG) reference standard, as well as decision coverage under deferral.

**Results:**

Agreement between real-world decisions and AI recommendations was 83.2% (sensitivity 74.7%, specificity 88.0%). Against the double-senior-graded reference standard (DSG), accuracy in the full analysis set (FAS), analyzed at the examination level, was 85.5% (sensitivity 77.2%, specificity 90.4%). Using an empirically optimized DAS threshold, accuracy increased to 88.6% (sensitivity 78.5%, specificity 94.9%) in the eligible retreatment-decision set (ERDS). Application of a deferral policy (“safety zone”, DAS 33–64) resulted in automated recommendations for 78.4% of eligible examinations, while 21.6% were deferred due to intermediate DAS values; among examinations with an automated recommendation, accuracy was 92.3% (sensitivity 83.2%, specificity 97.5%). Most misclassifications involved subtle IRF/SRF and SHRM as identified by the reading center and tended to be underestimated by the AI.

**Conclusion:**

The evaluated OCT-only AI decision-support output showed substantial agreement with routine retinal-specialist retreatment decisions in a real-world teleophthalmology workflow, particularly when intermediate Disease Activity Scores were deferred to human review. However, false-negative cases and context-dependent discrepancies highlight that the system should support, not replace, clinician judgement. Prospective multicenter validation using longitudinal and multimodal input data is required before broader workflow integration can be recommended.

## Introduction

Neovascular age-related macular degeneration (nAMD) is a leading cause of visual impairment among the elderly population in industrialized nations. With increasing life expectancy, its global prevalence and healthcare burden are expected to rise substantially (1). Although timely detection and anti-VEGF therapy can preserve vision, adherence to monitoring and treatment schedules remains suboptimal in real-world practice (2), particularly in decentralized regions with limited access to retinal specialists (3).

Artificial intelligence (AI) and deep learning (DL) systems have emerged as promising tools to improve diagnostic workflows and treatment decision-making in ophthalmology (4, 5). Trained on large datasets of optical coherence tomography (OCT) images, these algorithms can automatically detect biomarkers of disease activity - such as intraretinal and subretinal fluid or pigment epithelial detachment – with performance comparable to expert graders (6). Deep learning-based therapy planning support (TPS) systems have recently been developed to assist clinicians in evaluating disease activity and guiding treatment decisions in nAMD (7). The deepeye^®^ algorithm is the first CE-marked deep learning-based TPS tool designed for the automated analysis of OCT scans to assess disease activity in nAMD (8). By rapidly analyzing large OCT volumes and highlighting disease activity, AI-based TPS tools have the potential to standardize care pathways and reduce interobserver variability, thereby mitigating workload pressures in settings with limited specialist capacity. Nonetheless, their clinical applicability is constrained by data availability and the absence of contextual reasoning inherent to human assessment.

In teleophthalmology, retreatment decisions are often made under time constraints and with variable availability of longitudinal context. An OCT-based AI output that summarizes disease activity from a single current scan may therefore serve as decision support for triage and prioritization. Accordingly, the aim of this study was to evaluate real-world agreement between routine clinical management decisions and an OCT-only AI decision-support output, and to quantify the impact of an uncertainty deferral (“safety zone”) strategy on decision coverage and reliability.

## Methods

### Study design and setting

This retrospective single-center clinical usability study evaluated a European conformity (CE)-marked Therapy Planning Support (TPS) software (deepeye Medical GmbH, version 1.2) for automated OCT analysis in neovascular age-related macular degeneration (nAMD). The evaluated software version was locked before analysis. The algorithm generates a continuous Disease Activity Score (DAS, 0–100) and a binary activity recommendation based on OCT biomarker analysis. AI outputs were compared with real-world clinical retreatment decisions, and discordant cases underwent masked senior reading-center adjudication to establish a reference standard. Exploratory implementation analyses assessed alternative decision thresholds and an uncertainty deferral strategy (“safety zone”); restricted analyses are reported only for transparency and were not used for the primary endpoint. The study adhered to the Declaration of Helsinki and ICH-GCP (E6) and was approved by the Ethics Committee of the Medical Association of Westphalia-Lippe and the Medical Faculty of the University of Muenster (2025-946-f-S).

### Endpoints

The primary endpoint of this study was the agreement between the AI-based therapy planning support system and the real-world clinical retreatment decision made during routine teleophthalmology care. This endpoint was chosen because it reflects the intended clinical use of the AI system, captures the complexity of real-world decision-making, and represents the comparator that would be relevant for clinical implementation. Primary endpoint analyses were performed in the full analysis set (FAS), including all examinations.

The secondary endpoint was the agreement between the AI system and the double-senior-graded full-information reference standard (DSG). This comparator provides an expert-verified, high-quality reference independent of real-world variability and was used to contextualize discrepancies and assess the upper bound of AI performance. Secondary endpoint analyses were also performed in the full analyze set (FAS).

Exploratory analyses were performed on eligible retreatment-decision set (ERDS), which excluded cases in which predefined clinical or guideline-based factors independent of OCT activity assessment were identified during reading-center adjudication. These analyses included performance using an empirically optimized DAS threshold and performance restricted to high-confidence cases outside the predefined uncertainty (‘safety zone’). Additional exploratory analyses evaluated performance within the information-matched subset (IMS).

### Data collection

This retrospective single-center study included spectral-domain OCT examinations from treated eyes of patients with neovascular AMD, acquired between 1 February and 11 August 2024 using the Spectralis OCT system (Heidelberg Engineering, Heidelberg, Germany). The study was conducted in a predominantly White European/Caucasian clinical population; race and ethnicity were not captured as structured variables in the anonymized retrospective export. Agent-specific anti-VEGF information was not reliably available in the anonymized retrospective export and was therefore not analyzed. Treatment agent, retreatment interval, treatment regimen, prior OCT examinations, BCVA, and clinical notes were available to the treating retinal specialist during routine care but were not available to the OCT-only AI system and were not used as AI inputs.

For each examination, retreatment need was determined during routine teleophthalmology care by an experienced retinal specialist as either intravitreal injection or watch-and-wait. This real-world clinical decision was based on the current OCT examination, previous follow-up imaging when available, BCVA, treatment history, and all relevant clinical information documented in the case notes.

### AI assessment

deepeye^®^ TPS (Therapy Planning Support) is an AI-based software designed to assist eye care specialists in treatment planning for patients with nAMD. This study was conducted using deepeye^®^ TPS version 1.2.0. The software successfully completed the medical device certification process and received CE marking in May 2025, after completion of the present study.

The deepeye^®^ TPS analyses only the current volumetric SD-OCT examination and does not receive prior OCT examinations, fundus images, visual acuity, treatment interval, anti-VEGF agent, treatment regimen, or clinical notes as input.

The deepeye^®^ TPS generates an AI report comprising: (1) a disease activity assessment based on a convolutional neural network (CNN), (2) an additional long-term treatment-need prediction module, and (3) visualization of nAMD-related biomarkers generated by a UNet3+-based segmentation model. The present study evaluated only the disease activity assessment and its derived Disease Activity Score (DAS); the treatment-need prediction module was not included in the analysis.

At a high level, the software implements multi-stage processing of 3D OCT images, applying the three pre-trained AI models stated above: Biomarker Segmentation, Disease Activity, and Treatment Need. Exact OCT processing steps, AI model weights, and implementation details are proprietary. The AI models are locked within the software.

The output of deepeye^®^ TPS is a report displaying representative OCT B-scans with the greatest extent of intraretinal fluid (IRF), subretinal fluid (SRF), and pigment epithelial detachment (PED), based on segmentation masks generated by the Biomarker Segmentation AI model. The report also shows the Disease activity prediction displayed as a percentage (0–100%), which is used in this study as the Disease Activity Score (DAS; 0–100). The deepeye^®^ TPS report is intended to support, but not replace, clinician decision-making.

The present cohort was used only for retrospective external real-world evaluation of the pre-trained software. No OCT examinations, reading-center labels, real-world clinical decisions, derived thresholds, or other study-specific data from the present cohort were used to train, fine-tune, update, or otherwise modify the AI model. No study-specific post-processing was introduced.

For the primary analysis, the binary recommendation (“inject” vs “watch-and-wait”) was derived from the continuous DAS using the manufacturer-defined default threshold (DAS ≥ 50 indicating treatment). This threshold was pre-specified during training of the Disease Activity AI model, and integrated into deepeye^®^ TPS software before analysis of the present dataset; it was not derived from the current cohort.

### Discrepancy adjudication

To evaluate performance, AI-based and clinician-based activity assessments were compared. Specifically, discrepancies between real-world and AI decisions were classified as follows: FN (False Negative): TPS assessed activity as low, while the clinician detected activity for IVI treatment. FP (False Positive): deepeye^®^ TPS detected activity, but the clinicians decided for “watch-and-wait”.

All discrepant cases underwent re-evaluation by three senior medical retina specialists (BHB, MGu, CL), each with more than 15 years of clinical retina experience and experience in structured OCT grading and clinical-study adjudication. Regrading followed the guidelines of the German Ophthalmological Society and affiliated societies (9). Graders were masked to the AI output and to the original routine treatment decision. Cases were reviewed at three predefined information levels: (a) current OCT only, (b) current plus prior OCT examinations, and (c) full clinical information. The full-information level was used as the double-senior-graded (DSG) reference standard. Disagreements were resolved in a consensus conference. In addition, 25 concordant cases were re-assessed to verify consistency. Potential causes of disagreement were categorized as IRF, SRF, PED, SHRM, BCVA < 0.05 (approximately worse than 20/400 Snellen equivalent), poor image quality, need for prior OCT comparison, or patient-/management-driven factors.

### Image quality evaluation

OCT image quality was assessed using the Spectralis “Thickness Map” function. The exported quality index was recorded for each scan. Artifacts such as eye motion, defocus, shadowing, and vignetting were documented (10). Image quality metrics were included in the study database for correlation analyses with AI performance.

### Analysis populations

Three analysis populations were considered: the full analysis set (FAS), the eligible retreatment-decision set (ERDS), and the information-matched subset (IMS).

The FAS included all examinations and reflects routine clinical practice.

The ERDS was derived from the FAS using predefined classification criteria established during reading-center adjudication. Cases in which the reading-center adjudication identified predefined clinical or guideline-based factors independent of OCT activity assessment were excluded from the ERDS. These factors included schedule deviations, therapy refusal, BCVA <0.05, and non-nAMD pathology.

The IMS was derived from the ERDS and further excluded cases in which the reading-center adjudication relied on information unavailable to the OCT-only AI system or on image conditions outside the model’s intended operating characteristics. These included poor image quality, the need for comparison with prior OCT examinations, and the need for additional clinical history. The IMS was used exclusively for exploratory analyses and to provide an information-matched benchmark.

### Statistical analysis

No *a priori* power calculation for superiority was performed because the primary aim was to estimate agreement and operating characteristics with adequate precision. Statistical analyses were performed in R (version 4.5.0; R Foundation for Statistical Computing, Vienna, Austria). Agreement between the different raters (human or AI) and the DSG reference standard was evaluated using confusion matrices. Diagnostic performance metrics (accuracy, sensitivity, and specificity) were calculated on the examination level, reflecting the unit at which retreatment decisions were made in routine teleophthalmology practice. Because repeated examinations and bilateral eyes were available for some patients, 95% confidence intervals were estimated using patient-level cluster bootstrapping with resampling of patients and inclusion of all corresponding examinations in each bootstrap sample. Agreement and diagnostic performance were summarized using Cohen’s kappa, sensitivity, specificity, positive predictive value, negative predictive value, and 95% confidence intervals. The distribution of examinations per patient, eye-level characteristics, and the proportion of bilateral cases are reported in the cohort description.

Receiver operating characteristic analysis was performed using the continuous DAS, and the area under the curve (AUC) was calculated. McNemar’s test was used to assess asymmetry between discordant classifications.

The empirically optimized DAS threshold was evaluated as an exploratory calibration analysis. To reduce optimism from estimating and evaluating the optimized threshold on the same dataset, repeated patient-grouped 5-fold cross-validation (20 repetitions) was performed, ensuring that examinations from the same patient were not split between training and validation folds. The manufacturer-defined DAS ≥50 threshold served as the pre-specified threshold integrated into the software. Exploratory analyses additionally evaluated an empirically optimized threshold and an uncertainty deferral policy (‘safety zone’), in which intermediate-confidence cases were routed to human review.

To model the relationship between DAS and the probability of intravitreal injection, Bayes’ theorem was used to estimate the conditional probability P(IVI | DAS) based on the observed DAS distributions for IVI and watch-and-wait cases together with the empirical class prevalence [see **Supplementary Methods** for details (11)].

## Results

### Study population and analysis sets

A total of 429 spectral-domain OCT examinations from 306 eyes of 247 individuals with nAMD were included (31% male, 69% female; mean ± SD age 83.1 ± 7.3 years). Fifty-nine patients contributed bilateral eyes. The median number of examinations per patient was 1 (IQR (1, 2), range (1, 6)), and the median number of examinations per eye was 1 (IQR (1, 2), range (1, 4)). Ethnicity was predominantly Caucasian. Two eyes (0.7%) were treatment-naive, whereas 99.3% had previously received anti-VEGF therapy for a mean duration of 4.3 ± 3.3 years. BCVA was 0.52 ± 0.37 logMAR at the eye level.

### Primary endpoint: AI vs real-world clinical decisions (on FAS)

**Figure 1:**
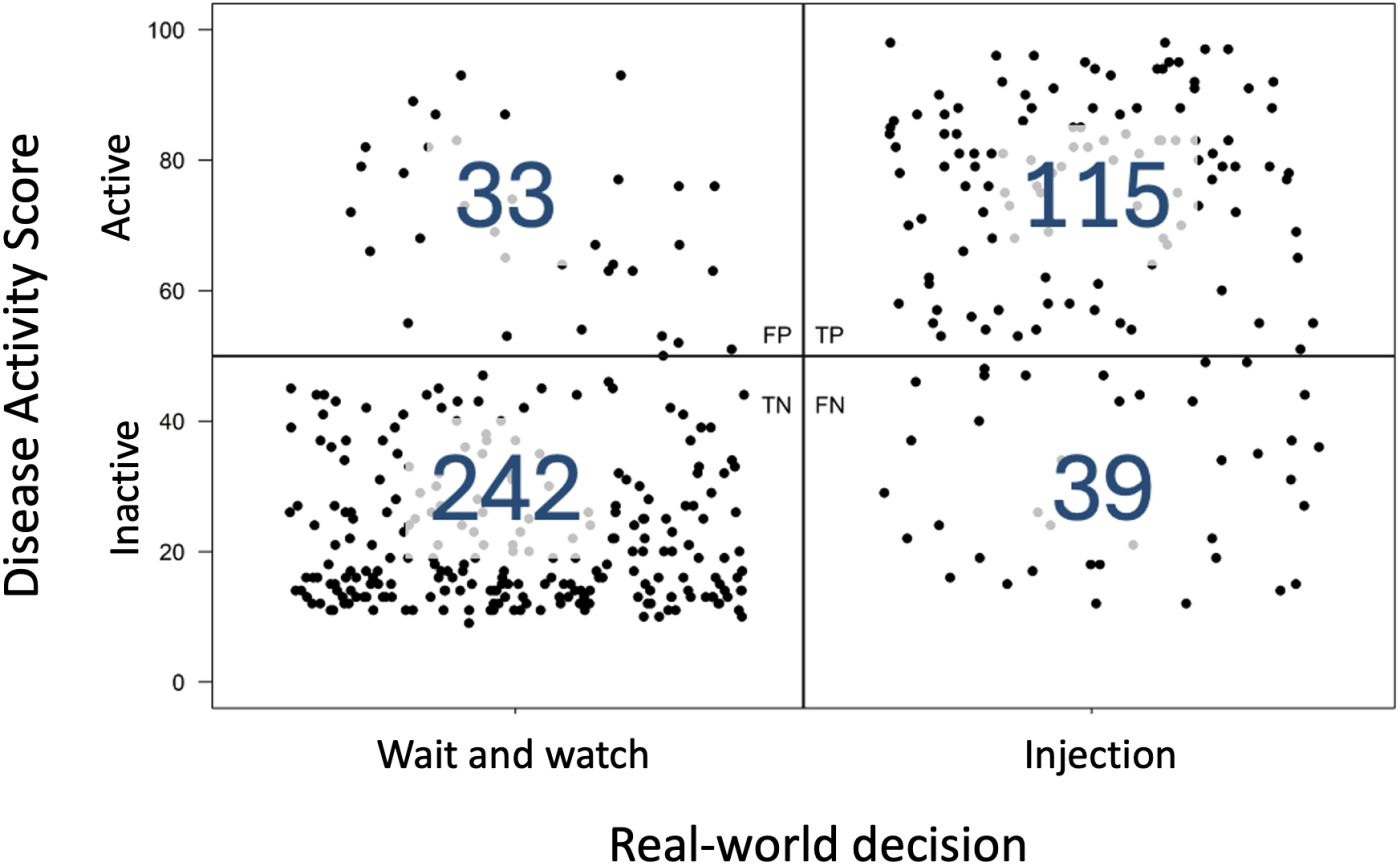
Comparison of AI-based and real-world treatment decisions in 429 cases of neovascular AMD. Concordance and discordance are shown based on disease activity scores (vertical axis, 0 – 100) versus clinical decisions (horizontal axis: “Watch-and-Wait” vs. “Injection”). Each quadrant illustrates the distribution of true positives (TP), false positives (FP), true negatives (TN), and false negatives (FN). The AI achieved an accuracy of 83.2%, sensitivity of 74.7%, and specificity of 88.0%.

Using the pre-specified manufacturer-defined DAS threshold of ≥50, corresponding to a recommendation for intravitreal injection, the deepeye^®^ TPS output showed substantial agreement with real-world clinician retreatment decisions. Out of 429 evaluated cases, 357 (83.2%; CI 79.7 – 86.7) showed agreement between AI recommendations and real-world clinical assessments, including 115 true positives, 242 true negatives, 33 false positives, and 39 false negatives. The resulting sensitivity was 74.7% (115/154; CI 66.4–81.7) and specificity was 88.0% (242/275; CI 83.8–91.8). The positive predictive value (PPV) reached 77.7% (115/148; CI 70.8–84.5), while the negative predictive value (NPV) was 86.1% (242/281; CI 82.0–90.1) (**Table 1**; **Figure 1**). Overall diagnostic agreement was substantial, with a Cohen’s κ of 0.63, and the area under the receiver operating characteristic curve (AUC) was 0.92. McNemar’s test revealed no significant asymmetry between discordant pairs (χ² ≈ 0.35, p ≈ 0.56), confirming balanced disagreement between the AI and clinical reference assessments.

**Table 1:** Diagnostic performance of deepeye^®^ TPS for retreatment decision support across different reference standards, thresholds, and datasets.

Analysis	Dataset	TP	TN	FP	FN	Accuracy (CI)	Sensitivity (CI)	Specificity (CI)
AI vs RW decisions	FAS (n=429)	115	242	33	39	83.2% (79.7–86.7)	74.7% (66.4–81.7)	88.0% (83.8–91.9)
AI vs DSG (predefined threshold)	FAS (n=429)	122	245	26	36	85.5% (82.0–88.9)	77.2% (69.5–84.1)	90.4% (86.3–94.0)
AI vs DSG (optimized threshold)	FAS (n=429)	124	244	27	34	85.8% (82.3–89.2)	78.5% (70.8–85.1)	90.0% (85.9–93.7)
AI vs DSG (optimized threshold)	ERDS (n=412)	124	241	13	34	88.6% (85.1–91.8)	78.5% (71.2–85.1)	94.9% (91.4–98.0)
Information–matched benchmark	IMS (n=393)	124	241	2	26	92.9% (90.1–95.3)	82.7% (75.9–88.7)	99.2% (97.9–100)
Safety–zone implementation analysis	ERDS (n=323)	99	199	5	20	92.3% (89.2–94.9)	83.2% (75.6–89.5)	97.5% (95.2–99.5)

RW, real–world clinical retreatment decision; FAS, full analysis set; ERDS, eligible retreatment–decision set; IMS, information–matched subset; DSG, double–senior–graded reference standard; TP, true positive; TN, true negative; FP, false positive; FN, false negative; CI, confidence interval. All confidence intervals are 95% confidence intervals estimated using patient–level cluster bootstrapping.

### Secondary endpoint: AI versus DSG reference standard (on FAS)

Reading center grading was performed at three incremental levels of information: (a) single-OCT level (current OCT only), (b) OCT-history level (current and prior OCT examinations), and (c) full-information level (OCT history plus medical records). **Supplementary Table 1** summarizes the agreement and diagnostic performance of the senior graders, real-world clinical decisions, and the AI system relative to the double-senior adjudicated reference standard (DSG) on the FAS. The comparison between real-world clinician decisions and the DSG using the full-information level defined the clinical benchmark for AI evaluation, yielding 93.2% accuracy, 86.2% sensitivity, and 98.3% specificity.

DSG adjudication resulted in reclassification of 12 discordant cases and 1 concordant case compared with the original routine decision. Using the DSG reference standard and the manufacturer-defined DAS threshold, AI performance was 85.5% accuracy (CI 82.0–88.9), 77.2% sensitivity (CI 69.5–84.1), and 90.4% specificity (CI 86.3–94.0) (**Table 1**). The adjudicated causes of the remaining discordant cases are summarized in a separate analysis below.

### Optimization of the AI threshold using statistical modelling of disease activity

To explore calibration of the software’s AI output, we performed a statistical modeling of the relationship between the AI-generated Disease Activity Score (DAS) and the probability of receiving intravitreal injection therapy (*P_IVI_*). Modeling the relation between DAS and the estimated probability of intravitreal injection (P(IVI|DAS)) suggested an empirical threshold of 48.75, while treatment probabilities of 20% and 80% corresponded to DAS values of 33.3 and 63.8, respectively (**Supplementary Figure 1**). Applying this threshold to the ERDS yielded accuracy 88.6% (CI 85.1–91.8), sensitivity of 78.5% (CI 71.2–85.1), and specificity of 94.9% (CI 91.4–98.0).

Because the optimized threshold was derived from the present dataset, this analysis should be interpreted as exploratory. To assess potential optimism, repeated patient-grouped 5-fold cross-validation (20 repetitions) was performed. Across cross-validation iterations, optimized thresholds remained highly stable with a mean threshold of 49.07 ± 1.03. Cross-validated accuracy was 88.3% ± 3.6%, closely matching the apparent performance in the full dataset and suggesting limited overfitting of the threshold optimization procedure.

### Information-matched benchmark (IMS)

To estimate expected AI performance under information-matched conditions, we evaluated the information-matched subset (IMS), excluding cases in which the DSG adjudication relied on information unavailable to the OCT-only AI system or on image conditions outside the model’s intended operating characteristics. These exclusions comprised poor image quality (n=12), need for prior OCT comparison (n=6), and need for additional clinical history (n=1).

In the IMS, AI performance improved to 92.9% accuracy (CI 90.1–95.3), 82.7% sensitivity (CI 75.9–88.7), and 99.2% specificity (CI 97.9–100.0). All IMS-excluded cases remained included in the primary analyses; the IMS is presented solely as an information-matched benchmark. These findings illustrate the impact of information mismatch on apparent AI performance and support the importance of context-aware evaluation of OCT-only AI systems.

### Analysis of AI misclassifications based on fluid and structural biomarkers

**Figure 2:**
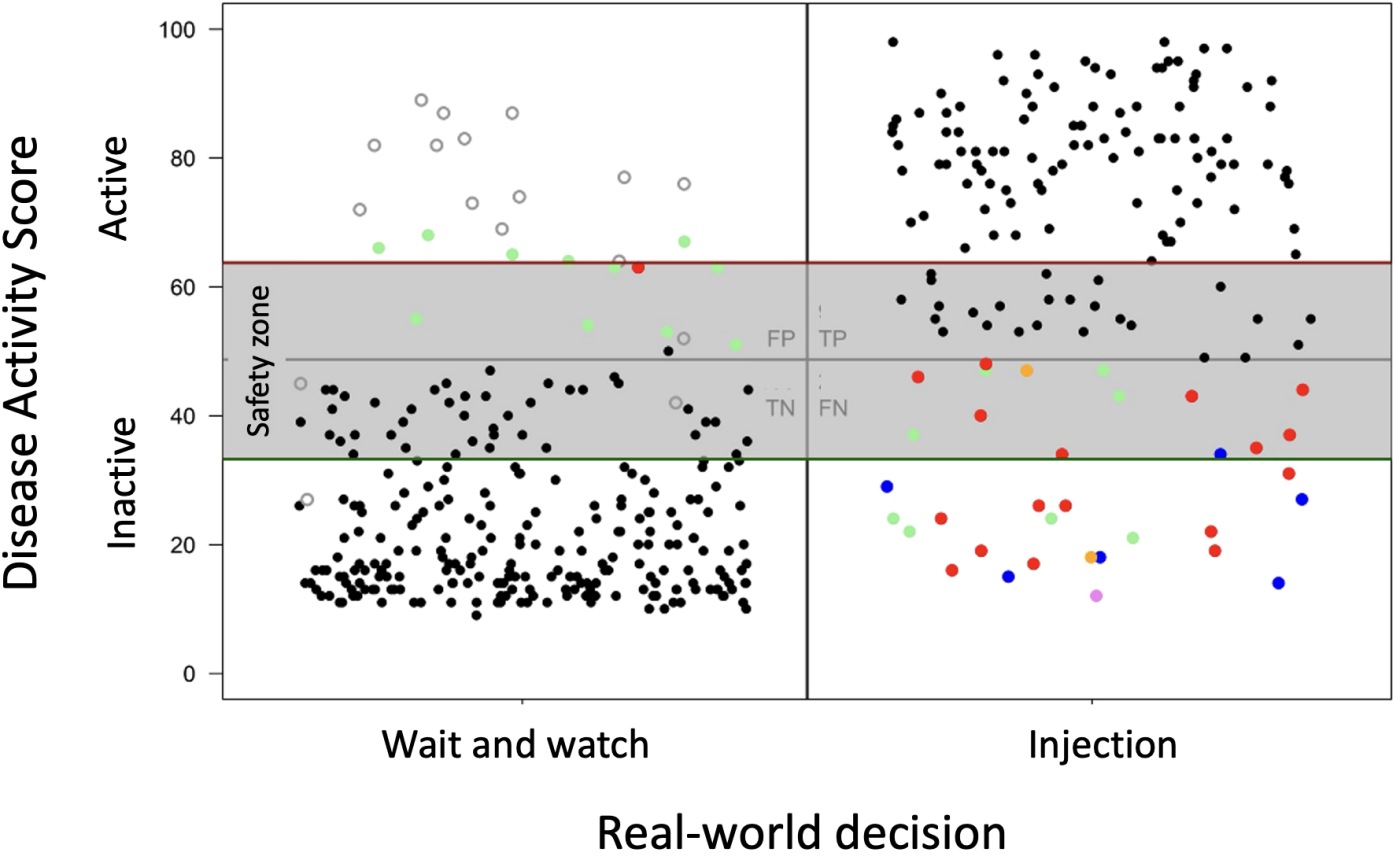
Distribution of AI Disease Activity Scores (DAS) and discordant cases. An uncertainty deferral policy (“safety zone”, DAS 33-64) defines intermediate scores for which no automated recommendation is issued and examinations are routed to human review. The quadrant plot illustrates examinations receiving an automated recommendation versus those deferred to human review, stratified by DAS thresholds and agreement with the DSG reference. Gray circles: examinations excluded from the ERDS due to predefined clinical or guideline-based factors; green dots: examinations excluded from the IMS; black dots: correctly classified; red dots: error due to IRF, blue dots: error due to SRF; orange dots: error due to SHRM; magenta dots: error due to PED.

**Figure 3:**
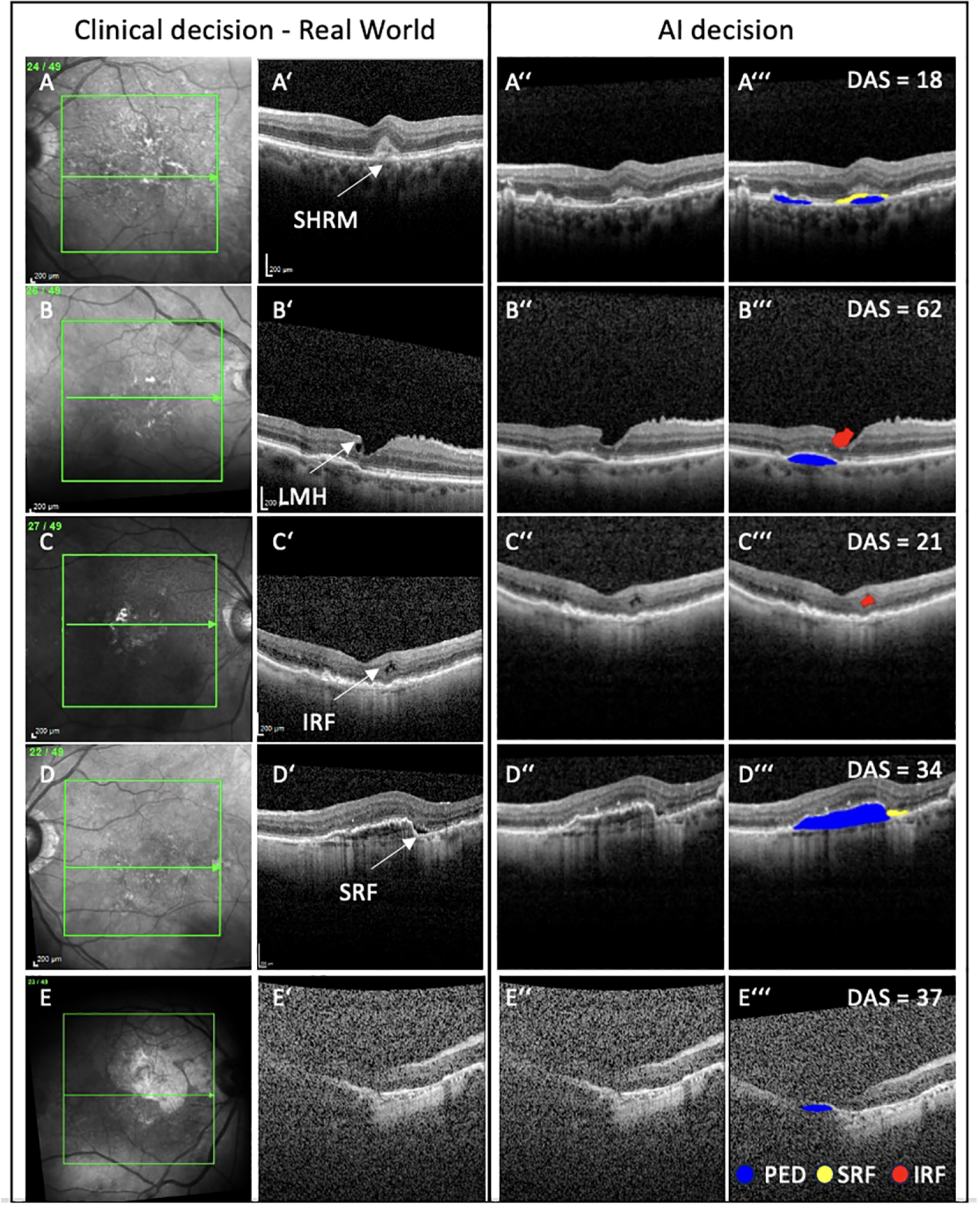
Examples of AI misclassification in comparison to real-world clinical decisions based on OCT imaging. Each row **(A-E)** presents a case with the real-world decision on the left and the corresponding AI assessment on the right. The real-world decision includes infrared fundus photography and expert grading of the OCT B-scan (columns **A′-E′**), indicating key disease features such as SHRM, IRF, and SRF. The corresponding AI assessment includes the same OCT B-scan **(A″-E″)** and the AI’s fluid segmentation overlays **(A‴-E‴)**, along with the computed Disease Activity Score (DAS). **(A)** Case with SHRM not featured by TPS report, resulting in low DAS (18). **(B)** Misclassification of a LMH as IRF, leading to overinterpreted DAS of 62. **(C)** IRF detected but underestimated by AI with low DAS (21). **(D)** SRF detected, but its relevance underweighted (DAS = 34). **(E)** Example of poor image quality (Heidelberg Quality Index = 11) preventing adequate evaluation.

For a better understanding of reasons for AI misclassification, the remaining discordant cases were analyzed based on specific morphological features identifiable in OCT imaging. Among the remaining discordant cases, 27 were attributed to clinically relevant OCT biomarkers that the deepeye^®^ TPS AI model either underestimated or misinterpreted (**Figure 2**): IRF (n=18), SRF (n=6), PED (n=1), and SHRM (n=2). These misclassified cases were located primarily in the false-negative quadrant, indicating that the AI assigned low disease-activity scores despite the reading-center decision to inject. These cases illustrate that subtle or ambiguous OCT findings, including small IRF pockets, shallow SRF, or atypical SHRM patterns, may be difficult to classify from a single OCT examination alone and therefore represent clinically relevant scenarios for human review or uncertainty-based deferral. **Figure 3** summarizes typical scenarios in which AI fails to recognize or accurately assess disease-relevant biomarkers, particularly in cases involving subtle fluid or reflectivity patterns, and highlights the impact of image quality on AI performance. These findings emphasize the need for further refinement of the evaluated AI algorithm in recognizing nuanced fluid compartments and structural biomarkers and support the inclusion of expert-verified annotation during training to reduce clinically relevant misclassifications.

### Exploratory implementation analysis: safety zone (on ERDS)

We evaluated an uncertainty deferral policy (“safety zone”, DAS 33–64), for which no automated recommendation was issued, and cases were routed to human review. This range corresponds to an estimated probability of intravitreal injection between 20% and 80%, reflecting intermediate disease activity where automated recommendations are less reliable. Under this deferral policy, the system issued an automated recommendation in 323/412 examinations (decision coverage 78.4%), while 89/412 (21.6%) were deferred due to intermediate DAS values.

Among examinations receiving an automated recommendation, accuracy was 92.3% (CI 89.2–94.9), sensitivity 83.2% (CI 75.6–89.5), and specificity 97.5% (CI 95.2–99.5) (**Table 1**). These findings represent a coverage-reliability trade-off rather than performance improvement through exclusion.

Most discordant cases clustered within the intermediate DAS range indicating that borderline scores were a major source of disagreement. Notably, 36 of 61 misclassifications (59.0%) occurred within the safety zone, indicating that borderline DAS values were a major source of disagreement (**Figure 2**). By explicitly deferring intermediate-confidence examinations to human review and limiting automated recommendations to high-confidence scores, agreement with the DSG reference was higher among examinations for which the system issued a recommendation. This “no-decision” approach may support safer integration of AI-based decision support into teleophthalmology workflows by reducing the risk of automated recommendations in uncertain cases.

## Discussion

This study evaluated an OCT-based AI decision-support output for nAMD activity triage within a real-world teleophthalmology workflow. We observed substantial agreement between AI recommendations and routine clinical retreatment decisions, and agreement was highest in examinations for which the system issued a recommendation under an uncertainty deferral (“safety zone”) strategy. Because the system intentionally operates on the current OCT volume without longitudinal clinical context, these findings should be interpreted as evaluation of an OCT-based activity signal rather than a full treat-and-extend planning tool.

The baseline diagnostic accuracy of 83.2% and AUC of 0.92 in this study are consistent with prior work on deep learning systems for retinal disease assessment. DeepMind’s Moorfields model achieved AUCs above 0.94 for macular pathology detection (12), while other OCT-based classifiers for nAMD differentiation similarly report accuracies exceeding 0.9 (13, 14). Recent studies have extended this to disease activity assessment and found AI-based fluid detection comparable or superior to expert grading in the HAWK and HARRIER datasets, achieving accuracies around 0.8 – 0.9 (15, 16). Most of these evaluations, however, were based on retrospective or tightly controlled clinical trial data, limiting applicability to everyday clinical practice. Notably, Potapenko et al. prospectively validated an AI model using temporal OCT comparison between visits - achieving autonomous, expert-level decisions in ~92% of cases. Interestingly, the addition of fundus photographs did not improve the AI model in this study, presumably because bleeding also causes morphological changes in the OCT (17). Liakopoulos et al. evaluated CNV activity and reported agreement between reading center graders and clinical practitioners in 74.9% of 2286 OCT scans (18). In comparison, the Disease activity AI model assessment integrated within the deepeye^®^ TPS achieved an AUC of 0.842 for nAMD activity. This model was developed using a real-world dataset of 42,815 OCT scans and validated on 4,461 OCT (19).

It is important to note, that so far published OCT-AI models in AMD have often focused on biomarker segmentation, diagnostic classification, or progression prediction rather than same-visit retreatment triage. Fluid-segmentation systems and benchmark challenges such as RETOUCH/RetiFluidNet primarily evaluate pixel- or compartment-level detection of IRF, SRF, and PED, whereas other AMD models address diagnostic classification, macular atrophy, or future disease evolution. The work of Lim et al. summarizes several different deep learning paradigms reported in the literature for retinal fluid segmentation in OCT images, with a mean IRF DICE score of 76.5 (range: 69.0 to 90.9), mean SRF DICE score of 82.1 (range: 58.8 to 95.3), and mean PED DICE score of 80.5 (range: 69.4 to 95.8) (20). The Biomarker segmentation AI model integrated within the deepeye^®^ TPS achieved pixel-level retinal fluid detection performance with DICE scores of 0.718 (AUC 0.859) for IRF, 0.701 (AUC 0.894) for SRF, and 0.729 (AUC 0.775) for PED (19).

These endpoints are related but not directly interchangeable with the present evaluation of a locked therapy-planning-support output against real-world treatment decisions and a senior-adjudicated reference standard. Consequently, direct numerical comparison should be interpreted cautiously. Future work should evaluate therapy-planning systems against shared external benchmarks when suitable datasets and task-matched endpoints become available.

A key methodological feature of this study was the double-senior grading (DSG) adjudication, which corrected 13 cases – underscoring that some discordance reflects clinical subjectivity rather than AI error. The DSG-defined benchmark (accuracy 93.2%, sensitivity 86.2%, specificity 98.3%) provided a senior-adjudicated reference for contextualizing AI performance and aligns with recommendations for expert-informed reference standards (21). Moreover, identifying an empirically optimized disease activity score (DAS) threshold at 48.75% improved classification balance and supports adaptive thresholding as best practice in clinical AI calibration (22). The empirically estimated threshold of 48.75 was close to the manufacturer-defined DAS threshold of 50 and is best interpreted as an exploratory calibration finding rather than as an independently validated alternative threshold. The manufacturer-defined threshold remains the primary pre-specified operating point in this evaluation. DAS calibration may be useful for future implementation planning; however, independent external validation is required before any threshold modification can be recommended.

Our detailed analysis of misclassified cases revealed that the AI tended to underestimate subtle intraretinal and subretinal fluid, consistent with known limitations in OCT segmentation networks (23, 24). Additionally, the AI operated on single time-point OCT images without access to longitudinal imaging, visual acuity data, or treatment history. Consistent with this interpretation, performance increased further in the information-matched subset, suggesting that part of the observed discordance reflected information unavailable to the OCT-only AI system rather than incorrect interpretation of the available OCT data. These findings support the importance of multimodal data integration and contextual information for accurate disease activity assessment, as previously noted by Ting et al. (25) and more recently by Schmidt-Erfurth et al. (26), who stress the importance of contextual information for accurate disease activity assessment. Furthermore, reduced OCT image quality negatively affected performance, in line with prior observations that signal strength impacts fluid detection (27). Such uncertainty-aware approaches reflect growing regulatory emphasis on model confidence estimation and human oversight (28–30).

To improve the reliability of AI-driven decisions, we introduced a “safety zone” that deferred cases with intermediate DAS values (33 – 64). Among examinations receiving an automated recommendation, accuracy versus the DSG reference was 92.3% (95% CI 89.2–94.9) and specificity to 97.5% (95% CI 95.2–99.5), approaching the benchmark of clinician-DSG agreement (93.2%). Uncertainty-aware AI design is increasingly advocated to avoid overconfidence in borderline predictions and support clinical safety. Such approaches are also in line with current recommendations by regulatory bodies like the FDA and the European Medicines Agency, which emphasize human oversight and uncertainty thresholds in AI decision support systems (29).

These results underscore the potential of AI to assist therapeutic decision-making in nAMD, particularly in ophthalmology or capacity-limited settings. Prior studies have shown that AI integration can improve access, reduce interobserver variability, and streamline workflows (21, 25). However, clinical deployment requires structured governance frameworks encompassing uncertainty thresholds, threshold calibration, and expert override mechanisms. Future work should focus on multimodal input integration (e.g., BCVA, symptom data), enhanced biomarker detection (SHRM, small IRF), and multi-center validation across diverse imaging systems and populations (31).

This study has several strengths. It evaluated an AI-based therapy planning support algorithm in a real-world teleophthalmology setting using robust reference standards through double expert grading. The detailed analysis of misclassified cases and the introduction of a “safety zone” framework for uncertainty handling provide practical insights for improving AI reliability and clinical trustworthiness. Nonetheless, several limitations require emphasis. First, the study was retrospective and single-center, and all examinations were acquired using Spectralis OCT; therefore, performance may differ across OCT devices, scan protocols, clinical workflows, populations, and disease phenotypes. Second, the primary analysis was performed at the examination level, reflecting routine teleophthalmology retreatment decisions. Because repeated visits and bilateral eyes introduce within-patient correlation, confidence intervals were estimated using patient-level cluster bootstrapping. Although this approach accounts for intra-patient dependency, residual within-patient correlation cannot be fully excluded. Third, the evaluated AI output was restricted to the current OCT volume and had no access to prior OCT scans, BCVA, treatment interval, anti-VEGF agent, or clinical notes. This design intentionally reflects an OCT-based activity signal but limits recognition of trajectory-dependent or context-dependent retreatment decisions. Fourth, the empirical threshold and safety-zone analyses were exploratory and require independent validation. Fifth, long-term patient outcomes, workflow effects, and clinician behavior under prospective AI-assisted care were not assessed. Finally, detailed anti-VEGF treatment information and some subgroup-specific variables were not available in the anonymized dataset, limiting the evaluation of treatment-specific subgroup effects. These limitations temper the interpretation of the findings and underline the need for prospective multicenter validation (32, 33).

In conclusion, this real-world evaluation suggests that an AI based OCT-input-only therapy-planning-support software can provide clinically meaningful decision support for nAMD activity triage in teleophthalmology, particularly when intermediate DAS values are deferred to human review. However, persistent false-negative cases, image-quality limitations, and decisions requiring longitudinal or clinical context demonstrate that the system should support, not replace, clinician judgement. Prospective multicenter studies across OCT devices, patient populations, and clinical workflows are required to determine safety, workflow impact, and patient outcomes before broader implementation.

## Data Availability

De-identified data may be available from the corresponding author upon reasonable request. Requests to access these datasets should be directed to Clemens Lange, clemens.lange@augen-franziskus.de.
