# Supplementary material for: Real-world evaluation of an OCT-based AI decision-support system for neovascular AMD activity triage in teleophthalmology
Source: Front Ophthalmol (Lausanne). 2026 Jul 1;6:1870572. doi: 10.3389/fopht.2026.1870572 (PMC13368572; doi:10.3389/fopht.2026.1870572)
Supplement: Supplementary file 1 [file DataSheet1.docx]

**Supplemental methods**

To estimate the probability of intravitreal injection as a function of the Disease Activity Score (DAS), we modeled the conditional probability $P(\mathrm{IVI}\mid\mathrm{DAS})$using Bayes’ theorem and the observed DAS distributions in IVI and watch-and-wait cases. Confidence intervals were estimated using the Clopper–Pearson method with additional regularization to stabilize estimates at the distribution margins.

For practical estimation of P(IVI∣DAS=s), observations within a local DAS neighborhood around s were considered. To obtain a sufficient number of samples, all cases with DAS value x within a margin δ around s were included, i.e. $x\in[s-\delta,\text{ }s+\delta]$. The choice of $\delta$ induces two opposing effects:

(1) A larger $\delta$increases the number of observations and therefore reduces the width of the confidence interval.

(2) At the same time, a larger $\delta$yields a less homogeneous subset, resulting in greater variability of $P_{\mathrm{IVI}}(x)$.

To balance these effects, we computed the mean $\mu_{s}(\delta)$and standard deviation $\sigma_{s}(\delta)$of $P_{\mathrm{IVI}}(x)$for all $x\in[s-\delta,\text{ }s+\delta]$. The adjusted conditional probability is defined as $\mu_{s}(\delta)$, and the adjusted confidence interval is given by

$$\mathrm{CI}(s):=[\mu_{s}(\delta_{\mathrm{opt}})-\varepsilon_{1}(\delta_{\mathrm{opt}})-\sigma_{s}(\delta_{\mathrm{opt}}),\text{ }\mu_{s}(\delta_{\mathrm{opt}})+\varepsilon_{2}(\delta_{\mathrm{opt}})+\sigma_{s}(\delta_{\mathrm{opt}})],$$

where $\varepsilon_{1}$and $\varepsilon_{2}$denote the lower and upper Clopper-Pearson confidence limits.
The optimal margin $\delta_{\mathrm{opt}}$was chosen to minimize the width of the adjusted confidence interval.

**Supplemental figure 1**
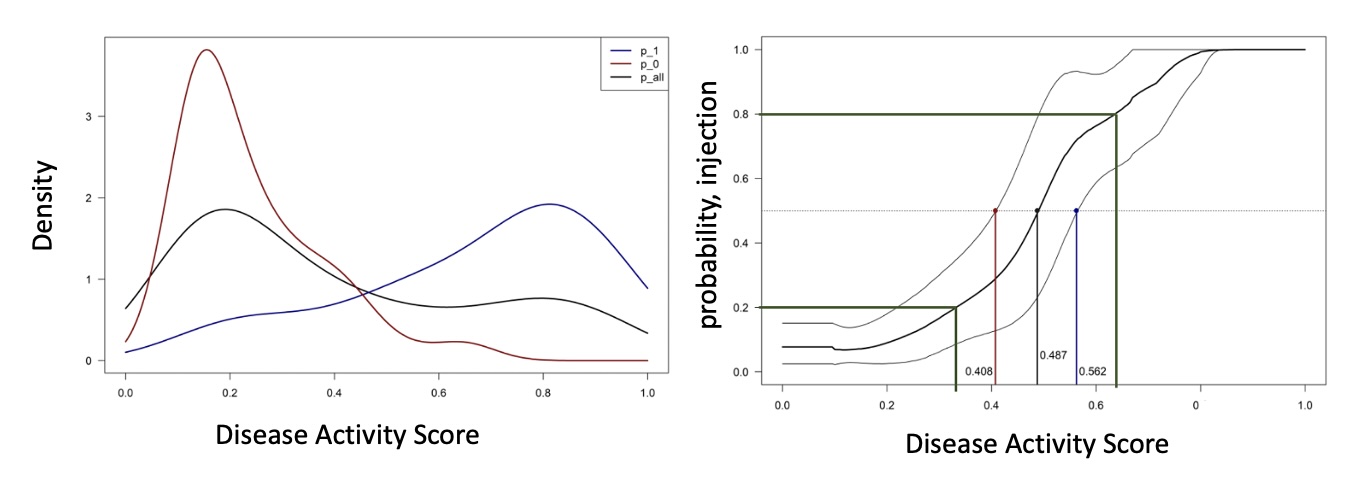


**Supplemental Figure 1:** Statistical modeling of the relationship between the AI-generated Disease Activity Score (DAS) and the probability of receiving intravitreal injection therapy ($P_{\text{IVI}}$), based on senior expert grading. Left panel: Density distributions of DAS for all cases (black), inactive cases (red), and active cases (blue). Right panel: Logistic regression curve showing the probability of IVI as a function of DAS with 95% vertical confidence interval. The empirically derived threshold for optimal decision separation is 0.487, distinguishing likely inactive (<48.7%) from likely active (>48.7%) disease. Dashed lines indicate example mappings: a $P_{\text{IVI}}$ of 20% corresponds to a DAS of 33.3; a $P_{\text{IVI}}$ of 80% corresponds to a DAS of 63.8.

***Supplemental Table 1***

| **Data Level** | **Rater** | **Kappa** | **95% CI** | **Acc.** | **Sens.** | **Spec.** |
| --- | --- | --- | --- | --- | --- | --- |
| one OCT | Senior 1 | 0.82 | [0.68; 0.96] | 91.0% | 81.9% | 99.3% |
| one OCT | Senior 2 | 0.92 | [0.82; 1.00] | 95.9% | 91.3% | 100.0% |
| one OCT | Senior 3 | 0.78 | [0.62; 0.95] | 89.2% | 86.0% | 92.1% |
| one OCT | Real-world | 0.75 | [0.61; 0.90] | 87.8% | 76.5% | 98.1% |
| one OCT | AI | 0.63 | [0.47; 0.80] | 82.0% | 65.8% | 96.8% |
| OCT History | Senior 1 | 0.89 | [0.78; 1.00] | 94.8% | 87.8% | 100.0% |
| OCT History | Senior 2 | 0.99 | [0.94; 1.00] | 99.3% | 98.3% | 100.0% |
| OCT History | Senior 3 | 0.78 | [0.61; 0.95] | 89.2% | 85.0% | 92.1% |
| OCT History | Real-world | 0.85 | [0.74; 0.97] | 92.9% | 85.7% | 98.3% |
| OCT History | AI | 0.69 | [0.53; 0.85] | 85.2% | 71.4% | 95.4% |
| Full Information | Senior 1 | 0.88 | [0.76; 1.00] | 94.1% | 86.0% | 100.0% |
| Full Information | Senior 2 | 0.98 | [0.92; 1.00] | 98.9% | 97.5% | 100.0% |
| Full Information | Senior 3 | 0.69 | [0.50; 0.89] | 85.0% | 85.9% | 84.4% |
| Full Information | Real-world | 0.86 | [0.74; 0.97] | 93.2% | 86.2% | 98.3% |
| Full Information | AI | 0.68 | [0.52; 0.84] | 84.9% | 71.2% | 95.0% |

***Supplemental Table 1:*** *Inter rater reliability (Cohen’s Kappa) of different rater compared to double-senior graded decision on different levels of information. To encounter the fact that only 25 of 357 cases without discordance are regraded, we projected the confusion matrix (ERDS analysis set) of the regraded cases to the set of all valid cases and computed Cohens Kappa, its 95% confidence interval (CI), Accuracy (Acc), Sensitivity (Sens.) and Specificity (Spec.). Remember that Real-world decision relies on full information and AI on one OCT.*
